# All Hands on Deck. Transforming the Health System Requires Innovation, Through Individual- and Diffusion Efforts: Comment on "Employee-Driven Innovation in Health Organizations: Insights From a Scoping Review"

**DOI:** 10.34172/ijhpm.8506

**Published:** 2024-07-13

**Authors:** Willem H. van Harten

**Affiliations:** ^1^Health Technology and Services Research, University of Twente, Enschede, The Netherlands; ^2^Psycho Oncology and Epidemiology, The Netherlands Cancer Institute, Amsterdam, The Netherlands

**Keywords:** Employee Driven Innovation, Diffusion, Scientific Impact, Digitalisation

## Abstract

Employee driven innovation (EDI) is essential in transforming hospitals and other providers, but the challenge is also to have impact on health systems as a whole. Usually a mix from top down measures and bottom up initiatives leads to an innovative culture. An important aspect is the innate difference between types of providers related to initiating, facilitating and rewarding innovation. Second the rewarding system within organisations but also in science and scientific journals. Especially nursing and other non-medical professions can be emancipated in this regard. Further there is a growing interdependence with digitalisation in all its forms and awareness of the related team effort is needed to actually realise innovative projects within a standing organisation. Lastly change the paradigm related to the spread of innovations from "not invented here" to "proudly copied from," create trust and organize collaboration between providers and spend sufficient attention to credible evidence on the effectiveness.

 Recently a paper published in this journal presented a scoping review on employee driven innovation (EDI).^1^ Growing numbers of aging patients that present with comorbidities, biotechnical developments and impressive pipelines of new expensive drugs, ever growing financial pressure and—not the least—workforce issues pose enormous challenges to health systems. It is unthinkable that this can be solved without social and technical innovation, and sometimes even transformation of many aspects of care provision. The legitimate position of mostly non-profit type of providers will only be respected and funded permanently if they find the drive to innovate and adapt to these new circumstances; in more market oriented systems providers additionally need to innovate to avoid the risk to loose their competitive edge.

 In research on innovation two main issues keep popping up over the years: How to actually and speedily innovate and transform your organization? and How to speed up the diffusion of sufficiently proven innovations through acceptance and implementation by organizations and/or their staff.^2,3^ Both issues play a role when dealing with and following up on EDI. Diffusion is an essential ingredient in healthcare as comparable providers exist in large numbers and it is extremely inefficient when every single organisation aspires to “invent a wheel” or suffers from the “not invented here” phenomenon. EDI is undoubtedly essential as frontline workers meet the patient/client in daily practice and understand core processes, but learning from other departments or divisions and copying form others is important for innovation of the system as a whole. Rogers description of factors that determine the diffusion throughout the system is still relevant, though a number of specific factors are presently determining its success and speed. In this commentary, suggestions for managerial-, policy-, and research priorities are provided.^4^

## Healthcare providers types differ in innovativeness.

 Academic centers have research and development as one of their core assignments and an intrinsic drive towards innovation. This however more often concerns the biomedical field and predominantly focuses on academic performance and biotechnical innovations. These are important for patients, but do not necessarily contribute to the solutions needing priority from a societal perspective. In large teaching hospitals there is a stronger focus on providing state of the art care, practice changing work is integrated in daily practice; though a personal observation, related to the size of operations, innovation is often structured and financed. Small sized providers are commonly strictly focused on performance and efficiency and lack dedicated staff and means to innovate under their own power. For policy-makers it is important to understand these differences as the diffusion of an innovation through the system has to cover all these types of providers. Differential approaches should thus be a topic of research.

## How to organize innovation and especially how to stimulate EDI, remains a pressing issue.

 Apart from the intrinsic drive of many professionals to improve and innovate their services, every provider has to innovate, to keep up with peers or to keep a competitive edge. Books have been written about how to achieve this, but leadership is always essential. To create an entrepreneurial and rewarding culture is not something that can be done overnight, it requires exemplary behaviour and acceptance of failures. Top down stimulation of innovation can be done by organizing tenders, innovation awards or outright investments. Facilitating innovation to flourish is really an issue and most authors agree that creating a free or experimental setting is crucial. Frontline workers often know best how to innovate but are also intertwined with current practice; line management most often—and understandably—has a strong focus on budget restrictions, quality assurance and safety management thus often suffocating initiatives without realizing it. Creating an innovation (support) group or unit that has sufficient room to neglect rules and regulations, is thus essential.

 A mix of stimulating top down and rewarding bottom up innovations is usually the most productive. As even within large organisations departments are not always aware of best practices, formal knowledge exchange and collaborative approaches seem underused and need to be reinforced and continuously stimulated. If you manage to establish an open exchange within large hospital systems and provide a formal collaborative structure it becomes easier to adopt innovations form others.^5^ For instance the mProve network of 7 large teaching hospitals in The Netherlands, representing almost 20% of the national hospital volume, decided to change the innovation paradigm: from “Not invented here” to “Proudly copied from….”^6^ The objective for management is to create a stimulating environment where EDI can flourish.

## Almost every innovation has a digital element.

 Presently, almost every element of a hospital’s operations has a digital component, be it Care Pathways, Operating Room or Intensive Care processes, Enterprise Resource Programs, Operations Improvement and Redesign, Value Based Health, or Multifunctional Patient Portals. As a consequence most innovations are digital (patient monitoring apps, artificial intelligence applications) use digital technology (remote monitoring or monitoring as a service) or at least have to be checked on patient safety and cybersecurity issues before being connected to the hospital information system or the electronic medical record (EMR). Massive attention for conferences such as Healthcare Information and Management Systems Society as well as resources provided underscore this.^7^ The information technology department thus becomes crucial in the speed with which innovations can actually be implemented. Maybe a freestanding system can be used for the first pilot phase of innovations, once scale and diffusion speed are needed for which adequate embedding within and connectivity with all relevant hospital IT systems are needed, the dependence on the IT department will grow.^8^ Stimulating EDI requires fostering a certain degree of digital and social competences and that aspect seems somewhat neglected in the paper of Cadeddu et al.^1^

## Rewarding research on innovation.

 Earlier I pointed out that biomedical innovation is inherent to academic institutions and teaching hospitals, this however mainly concerns the professional domains. Careers depend on publishing in preferably high ranking journals and next steps in terms of patent filing or other forms of exploiting intellectual property. In this domain the employee has innovation as part of his/her job. This does not show from the EDI literature search as “innovation” is seldom a keyword while publishing on biomedical trials and advances. Journals seek cutting edge research on treatments and biomedical innovations to boost their impact. Papers on social and process innovation receive less attendance in high ranking journals and journals focusing on nursing and non-medical professions usually have a lower impact. The authors note that papers in their review are often published in nursing journals. Organizational innovation, efficiently employing staff and workforce improvement and innovative forms of care provision are not easily published in high ranking journals. Our group managed to publish a series of papers on service improvement, benchmarking and digital innovations but seldom managed to publish in journals with an impact factor above 10.^9-13^ Rewarding papers on innovations that actually deal with the main societal issues confronting healthcare by publishing them in higher impact journals would assist in creating better conditions for adoption and more professional respect. The recently initiated New England Journal Catalyst Journal on Innovations in Care Delivery may become an exception.^14^

## Convincing others to adopt and copy.

 Successful diffusion is commonly very rewarding for the team or individual initiating an innovation, thus reinforcing EDI. Rogers and Greenhalgh have identified a number of factors that are influential in individuals or organisations deciding to adopt an innovation.^2,4^ One aspect that is repeatedly hampering innovations to spread and frustrates the innovator (in: not being copied) is the evidence or proven effectiveness. Claims of success are often insufficiently substantiated by firm evidence or peer reviewed publications. The research tradition in social and digital innovation is less strong, but also faces methodological challenges. Controlled trials are difficult to perform as evaluating organisational performance requires a number of alike units that are sufficiently comparable and still seldom reach sufficient power. Commonly case-, case-control studies, interrupted time series are used and seldom a randomized controlled trial is seen.^15,16^ However if we want to realize a shift from “not invented here” to “proudly copying from,” using more firm designs in research or agree beforehand on simple sets of evaluation indicators in a collaborative will surely assist diffusion.

 In digital health it is further important to distinguish between offline/in vitro and online/real-life type of pilot work. Especially the integration with the hospital’s EMR and information system is an issue. Can providers actually invest in systems that facilitate the digital (aspect of the) innovation, is there connectivity, is working through the EMR possible? These are aspects that can easily prevent an innovation from being adopted on a larger scale.^8^

## Conclusion

 Innovation is essential to face future health system challenges.

 Organisations should facilitate EDI according to their possibilities. The primary issue is to create a culture and infrastructure for innovation to thrive: Having fun and being rewarded, allow failure and proudly copying instead of inventing your own wheel are important aspects.

 Emancipating the nursing profession can aid in speeding up this field, especially in balancing EDI with organisational and system wide efforts.

 Diffusion is as important as innovation, so compelling and objective, sometimes scientific, evidence should accompany communication. Acceptance and copying are depending on motivated staff that feel professionally challenged to take action.

 Academic standing of social and technological innovation should be improved. Major journals should strive to publish other than strict biomedical papers, related to innovation in their defined scope.

## Ethical issues

 Not applicable.

## Competing interests

 Author declares that he has no competing interests.
